# Predicting FFAR4 agonists using structure-based machine learning approach based on molecular fingerprints

**DOI:** 10.1038/s41598-024-60056-z

**Published:** 2024-04-24

**Authors:** Zaid Anis Sherwani, Syeda Sumayya Tariq, Mamona Mushtaq, Ali Raza Siddiqui, Mohammad Nur-e-Alam, Aftab Ahmed, Zaheer Ul-Haq

**Affiliations:** 1 Dr. Panjwani Center for Molecular Medicine and Drug Research, International Center for Chemical and Biological Sciences, University of Karachi, Karachi, 75270 Pakistan; 2 H.E.J Research Institute of Chemistry, International Center for Chemical and Biological Sciences, University of Karachi, Karachi, 75270 Pakistan; 3 Department of Pharmacognosy, College of Pharmacy, King Saud University, P.O. Box. 2457, Riyadh, 11451 Kingdom of Saudi Arabia; 4 Department of Biomedical and Pharmaceutical Sciences, Chapman University School of Pharmacy, Irvine, CA 92618 USA

**Keywords:** FFAR4, Bayesian network algorithm, Structure-based machine learning, Molecular dynamics simulations, Drug screening, Biochemistry, Drug discovery, Molecular biology, Molecular medicine

## Abstract

Free Fatty Acid Receptor 4 (FFAR4), a G-protein-coupled receptor, is responsible for triggering intracellular signaling pathways that regulate various physiological processes. FFAR4 agonists are associated with enhancing insulin release and mitigating the atherogenic, obesogenic, pro-carcinogenic, and pro-diabetogenic effects, normally associated with the free fatty acids bound to FFAR4. In this research, molecular structure-based machine-learning techniques were employed to evaluate compounds as potential agonists for FFAR4. Molecular structures were encoded into bit arrays, serving as molecular fingerprints, which were subsequently analyzed using the Bayesian network algorithm to identify patterns for screening the data. The shortlisted hits obtained via machine learning protocols were further validated by Molecular Docking and via ADME and Toxicity predictions. The shortlisted compounds were then subjected to MD Simulations of the membrane-bound FFAR4-ligand complexes for 100 ns each. Molecular analyses, encompassing binding interactions, RMSD, RMSF, RoG, PCA, and FEL, were conducted to scrutinize the protein–ligand complexes at the inter-atomic level. The analyses revealed significant interactions of the shortlisted compounds with the crucial residues of FFAR4 previously documented. FFAR4 as part of the complexes demonstrated consistent RMSDs, ranging from 3.57 to 3.64, with minimal residue fluctuations 5.27 to 6.03 nm, suggesting stable complexes. The gyration values fluctuated between 22.8 to 23.5 nm, indicating structural compactness and orderliness across the studied systems. Additionally, distinct conformational motions were observed in each complex, with energy contours shifting to broader energy basins throughout the simulation, suggesting thermodynamically stable protein–ligand complexes. The two compounds CHEMBL2012662 and CHEMBL64616 are presented as potential FFAR4 agonists, based on these insights and in-depth analyses. Collectively, these findings advance our comprehension of FFAR4’s functions and mechanisms, highlighting these compounds as potential FFAR4 agonists worthy of further exploration as innovative treatments for metabolic and immune-related conditions.

## Introduction

FFAR4, also known as Free Fatty Acid Receptor 4 is a G-protein-coupled receptor (GPR120) found in humans. It is predominantly present in the gastrointestinal system, adipose tissue, and immune cells. FFAR4 plays a crucial role in mediating the effects of long-chain fatty acids, particularly omega-3 fatty acids, in various tissues. When activated by these fatty acids, FFAR4 triggers intracellular signaling pathways that regulate various physiological processes, such as glucose and lipid metabolism, inflammation, and insulin sensitivity^1^. Consequently, FFAR4 has garnered significant attention as a potential target for conditions like obesity, diabetes, and inflammatory disorders^2^. Understanding the functions and mechanisms of FFAR4 in humans may offer valuable insights into the development of novel treatments for metabolic and immune-related diseases.

The role of FFAR4 in regulating blood glucose levels has been established since long. FFAR4 activators are purported to be useful in treating type 2 diabetes^3^. In a recent study, several FFAR4 agonists were stimulated to release insulin from murine pancreatic islets and the resultant post-prandial glucose peak was found to be mitigated. In another study, a co-agonist small molecule at FFAR1 and FFAR4, enhanced the uptake of glucose by mouse adipose cells in diabetic models in rodents, as a result, insulin secretion was stimulated significantly and blood glucose levels were lowered^4^. In mice, blood glucose and insulin profiles were improved by a different co-agonist of FFAR1 and FFAR4. A significant finding in related studies suggests a release of glucagon-like peptide-1 (GLP), gastric inhibitory peptide (GIP), and muted ghrelin secretion by FFAR4 agonists as a result of insulin stimulation, which is attributed to the anti-glycemic effects^5^. People with higher insulin and lower HbA1c levels have higher amounts of FFAR4 in their pancreatic islets. It was also found that FFAR4-knocked-out mice are severely glucose intolerant. FFAR4 activators do not require free fatty acids, and can enhance insulin release and mitigate other atherogenic, obesogenic, pro-carcinogenic, and pro-diabetogenic effects attributed to free fatty acids^6^. This rationale has been the driving force to seek out potentially therapeutic FFAR4 agonists.

For this research, machine learning techniques based on molecular structures were used to screen compounds as potential FFAR4 agonists. In contrast to descriptor-based screening, which involves the calculation of numerous physicochemical descriptors submitted to machine learning algorithms for pattern recognition, the structure-based approach encodes molecular structures into bit arrays based on the specific location, atom type, and topology. These assembled bit arrays, representing the molecules' structures, are then subjected to machine learning algorithms to establish patterns for the selected compounds^7,8^. By presenting derived fingerprints about the structures of known activators, and inhibitors of FFAR4, and a decoy dataset, a training set was used to train our machine learning model. Bayesian network algorithm was used in this study as the fingerprints were encoded bit arrays and the Bayesian algorithm calculates the posterior probability of values appearing in the training dataset to conclude what ‘might be required in the structure for it to be an activator. The model so trained was used to filter compounds from a large screening dataset^9^. The shortlisted hits were proceeded to ADME prediction, Molecular Docking and post-docking analysis (to verify appropriate binding affinities and binding poses of the hits) and molecular dynamics of the Membrane-bound FFAR4—ligand complexes were carried out to document the stability of human FFAR4 with the hits.

## Materials and methods

### Structure based machine learning

Structure-based machine learning protocols were employed to find activators of Human FFAR4 (Free Fatty Acid Receptor 4), active compounds were retrieved (activators with documented EC50s) and inactive (inhibitors with documented IC50s) from the ChEMBL database^10^. A total of 467 active compounds and 1200 inactive compounds were downloaded in a catenated SDF format. Decoys were generated by inputting the SMILES of actives in the DUDE DECOYS webserver^11^ and randoms were taken from the ChEMBL Database. The ChemDes webserver^12^ was then used to calculate Morgan fingerprints of actives, inactive, decoys and random compounds.

The training set file was then put through WEKA software using BayesNET^13^ to generate our learning model. After evaluation of the best model via evaluation indices (performance metrics like kappa statistic, recall, precision, MCC, and F1 SCORE), the model that was deemed satisfactory was used to screen the test set. The test set was obtained by applying the Lipinski rule of 3, and calculating the Morgan fingerprints of all the compounds in the ChEMBL database, narrowing down the 6 million compounds in CHEMBL database to a total of 32,000 compounds. After screening through our learning model, a total of 693 compounds were shortlisted as actives from the screening database.

### Swiss ADME analysis

The Swiss ADME web server was utilized in order to assess the vital characteristics of the selected hits. These critical properties include physicochemical attributes, pharmacokinetic parameters, bioavailability considerations, permeability across the blood−brain barrier, adherence to Lipinski’s Rule of 5, synthetic accessibility, and the potential toxicity of small molecules. For the evaluation, the structural coordinates of the shortlisted hits were imported in 1D formats (SMILES notation) and were then submitted for an in-depth analysis via the server.

### Molecular docking

The structure of Human FFAR4 (Free Fatty Acid Receptor 4) was retrieved from RCSB PDB with the PDB ID-8ID3^14^. The protein preparation tool within Molecular Operating Environment (MOE v2019.01)^15^ was employed to optimize the protein structure for molecular docking, including processes such as assigning precise bond orders, removing redundant water molecules, applying terminal capping, supplementing any absent atoms, utilizing the AMBER99 force field, assignment of partial charges and minimization of energy etc. Additionally, any missing hydrogen atoms were added in accordance with pH 7's typical protonation state.

Triangle Matcher was employed as the main docking placement method for molecular docking, and AffinitydG was used as the placement scoring mechanism. The 50 resulting poses were refined using the "Induced Fit Receptor" method, and the top 10 poses determined by the GBVI/WSA dG technique were then subjected to additional study. In order to visualize, detect, and further evaluate protein–ligand interactions, the binding poses of the top-ranked compounds were examined using the Protein–Ligand Interaction Fingerprinting (PLIF) and Protein–Ligand Interaction Profiler (PLIP). In-depth analysis of the protein–ligand interactions and visualization of the structures and interactions are made possible by PLIP, which also serves to validate the docking data.

### Molecular dynamic simulation

Membrane Molecular dynamic simulations of FFAR4 with the 3 shortlisted compounds, with CHEMBL IDs CHEMBL2012662, CHEMBL1903952, CHEMBL64616 and Apo protein were so carried out using Amber22^16^. Since FFAR4 is a membrane receptor, the membrane protein–ligand systems were constructed via CHARMM-GUI webserver^17^. The protein was uploaded onto the server and subsequently, its transmembrane PDB was oriented within a lipid bilayer mimicking the typical constitution of a human cell membrane, comprising 95% POPC and 5% cholesterol. These systems were constructed to span 140 angstroms in both length and breadth. To simulate physiological conditions, TIP3P water was utilized along with the addition of ions in the form of KCl at a concentration of 0.15 mM, mirroring the typical molarity of human body fluids. Following this, the structural components including the protein, membrane, water, and ions were assembled, generating the system in PDB format. Using the CHARMM-GUI webserver, system files were prepared to undergo MD simulations in AMBER22. To ensure system stability, a brief 1 ns NPT ensemble minimization was conducted to alleviate any strains. Subsequently, the systems underwent 100 ns MD runs utilizing CHARMM36m force field^18^ while general Amber force field (GAFF) was employed for the ligand^19^.

Initially, the systems underwent conjugate gradient minimization and energy minimization using the steepest descent method with 2500 steps^20^. The protein was held in place using position constraints, and the procedure was repeated six times with a progressive reduction in the amount of restraint. At last, the imposed constraint was removed, and the systems underwent an additional energy minimization cycle. The systems were heated under a continuous volume, temperature (NVT) ensemble for 500 ps to reach the goal temperature of 300 K. Two iterations of the procedure were conducted, with a progressive reduction in positional constraints from 30 to 10 kcal mol. In the next steps, the equilibration was done for around 3.5 ns at constant pressure and temperature (NPT) ensemble. Finally, a production run lasting 100 ns for each complex was carried out using periodic boundary conditions. Thanks to isotropic position scaling and Langevin dynamics, the temperature and pressure were managed. Using the Particle Mesh Ewald (PME) technique, long-range electrostatic interactions were studied^21^. The SHAKE algorithm was utilised to restrict the interactions involving H atoms, and a cutoff of 10 Å was applied to determine the non-bonded interactions^22^. At a time step of two fs, the numerical integration was configured. VMD^23^ and the CPPTRAJ^24^ programme were used to examine the simulated trajectories. Root mean square deviation (RMSD), root mean square fluctuation (RMSF), and radius of gyration (RoG) are among the stability measures taken into consideration here. The donor and acceptor atoms’ cutoff distance for an H-bond was determined to be 3.5 Å, with a bond angle of 120. The percentage of the simulation run that H-bonds formed was used to compute occupancy showed in Table S3.

### Principal component analysis

The intricate data from MD Simulation underwent simplification via Principal Component Analysis (PCA) to condense the dimensions of the intricate data, aiming to unveil pivotal patterns and connections within the dataset. Its purpose extended to analyzing protein conformational changes and extracting meaningful insights from the intricate motions observed in MD trajectories. By aligning MD trajectories as part of the default settings, PCA was strongly grounded. The Essential Dynamics (ED) method was applied to compute eigenvectors, eigenvalues, and their projections by diagonalizing the matrix. Emphasizing the two principal components, the analysis was conducted using the PCA module within the MDAnalysis tools^25^. Additionally this principal components data were used to construct free energy profile of the simulated system.

### Free energy landscape

After evaluating the conformational landscape and the molecular motions investigated by the simulations, the GROMACS software package’s *gmx sham* plugin was used to construct the free-energy landscape^26^. Based on the first two primary components, the following equation shows the possible protein conformations during molecular dynamics simulations in relation to Gibbs free energy.

$$\Delta G= -{K}_{B}TlnP(P{C}_{1},{PC}_{2})$$

In this equation ${K}_{B}$ and $T$ are the Boltzmann constant and absolute temperature and $P\left(P{C}_{1},{PC}_{2}\right)$ is the probability distribution of the molecular system along with two principal components. The free energy landscape is a visual representation of the system’s energy distribution, that provides an insight into the kinetics and thermodynamic stability of the molecules, revealing various energy states and their associated conformational probabilities.

A flowchart detailing methodology workflow is available in in supplementary as in Fig. S1.

## Results and discussion

### Structure based machine learning

In the present study, the CHEMBL was used to assemble a training set of 437 FFAR4 actives (activators), in-actives (inhibitors), decoys (the molecules with the same physicochemical formula of actives but different topology and no activity at the receptor) and randoms. Chemopy Morgan fingerprints were calculated using CHEMDES webserver^27^ and all subsets were assembled into one training set file (Supplementary Information).

**Figure 1:**
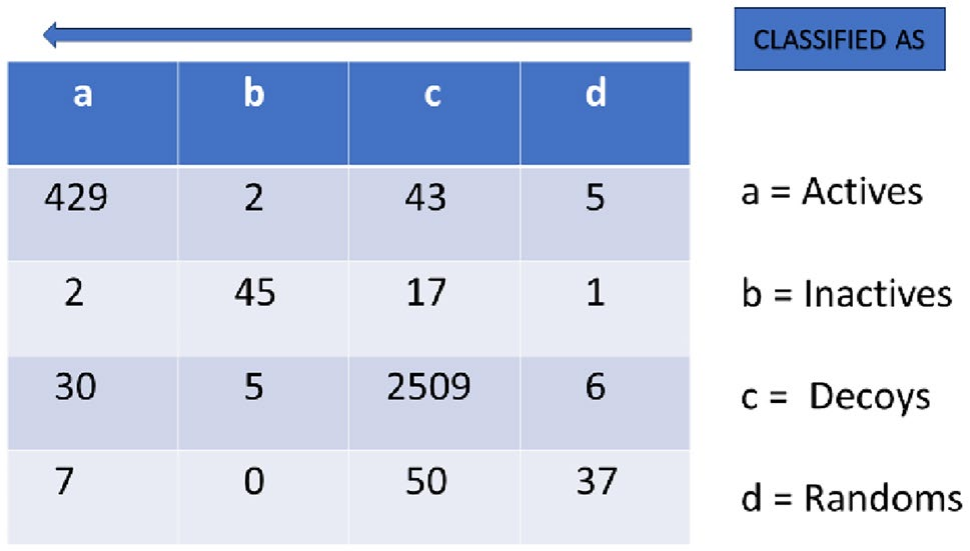
The confusion matrix for the best model trained.

The classification algorithm BayesNET which works on Bayesian network theory was chosen to derive a model as it is most suitable for data with bit arrays entries. Bayesian network algorithm takes the entries and calculates a posterior probability (the model so created is based on the preponderance of occurrence of similar entries and hence taken as features which mostly recur in a particular labelled compounds and hence likely to be essential for the behavior of this compound). The WEKA software based Bayesian network classification reported the best model with the kappa statistic of 0.7. The confusion Matrix resulting from BayesNET algorithm classification and resultant model is shown in Fig. 1.

Kappa statistic is the measure of predictability and generalizability of the model. It is a gauge of the extent to which a huge amount of data can be utilized to provide an accurate result i.e. it denotes the degree to which the study's data accurately reflect the factors under investigation.

It is possible to calculate Cohen's kappa using the following formula:

$$\kappa =\frac{{\text{Pr}}\left({\text{a}}\right)-{\text{Pr}}\left(e\right)}{1-{\text{Pr}}(e)}$$

where Pr(e) denotes chance agreement and Pr(a) is the actual observed agreement.

In other words, the kappa statistic of 0.821 indicates that the best model has the generalization ability (for a model applied to an external dataset with what accuracy it is likely to pick a true-active) of around 82%. Other evaluation metrics (F1 score, Mathew Correlation Coefficient (MCC), Precision vs Recall) are presented in table and speak of good predictive accuracy of the model. The detailed statistics of the model are shown in Table 1.

**Table 1:** Evaluation metrics attained by best trained model by BayesNET classifier algorithm employed on training set of known Actives, In-actives, Decoys and Randoms in Weka software.

Kappa statistic	Precision	Recall	F-Measure	MCC	Roc area	Precision area
0.821	0.944	0.947	0.944	0.843	0.977	0.470

**Figure 2:**
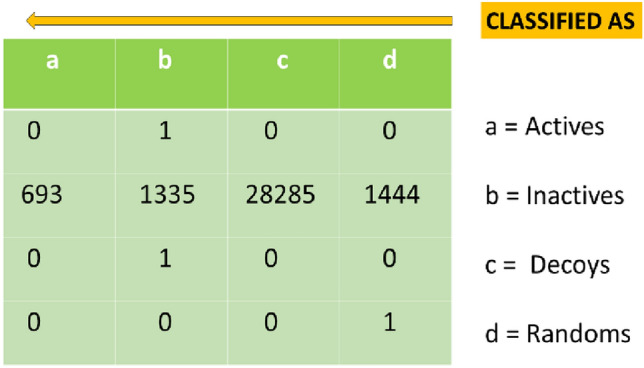
The sorted result of screening by applying the best trained model under the BayesNET algorithm. All the entries in the screening dataset were labelled in-actives at first. Total 693 compound were predicted to be actives.

After verification, this model was proceeded to screen the test set. The test set contained 32,000 compounds having applied Modified Lipinski Rule of 3 to 6 million compound in CHEMBL Database. These had Chemopy Morgan fingerprints calculated as per the training set. Upon screening this dataset, 693 compounds were picked up as actives as shown in Fig. 2.

### Molecular interactions

**Figure 3:**
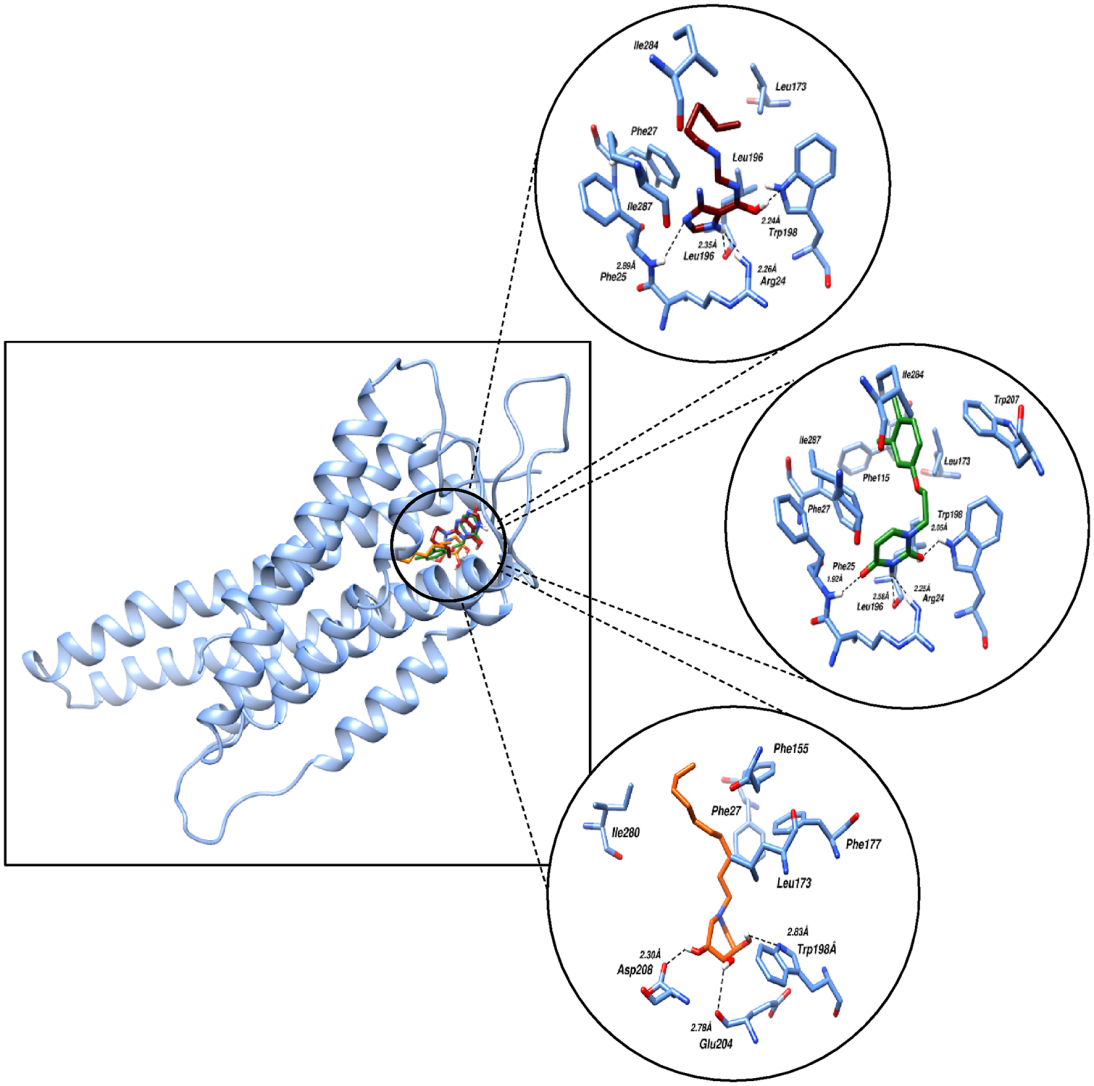
Molecular Interactions observed as a result of molecular docking of the three shortlisted compounds (Compound 1: Maroon, Compound 2: Green, Compound 3: Orange) with FFAR4. The interacting residues are represented as sticks, while black dotted lines represent the hydrogen bonds.

Molecular docking was applied to the 693 hits that came from structure-based screening using machine learning at the target protein's active site within the designated grid. When choosing the possible hits, docking interactions with important residues in the target site were evaluated. The MOE-implemented PLIF module was used to analyze the shortlisted compounds through protein–ligand interactions fingerprinting (PLIF) analysis. The major interactions between ligand and protein are identified using PLIF, which is regarded as a potent technique. The resulting hits were further narrowed down to 127. The compounds that were shortlisted had binding energies ranging from − 8.85 to − 4.0 kcal/mol. The Protein–Ligand Interaction Profiler (PLIP) was employed to examine the binding poses of the highest-ranked compounds, facilitating the visualization, identification, and in-depth analysis of protein–ligand interactions. Three compounds were finally shortlisted based on the interactions reported earlier^14^. Interactions of the compounds with the residues Leu196 and Trp198 of FFAR4 were commonly observed in case of all three complexes, while residues Phe25, Glu204, Asp208 also took part in hydrogen bonding. The PLIP interactions of the final 3 compounds shortlisted based on interactions with crucial residues are shown in Fig. 3.

### Swiss ADME

**Figure 4:**
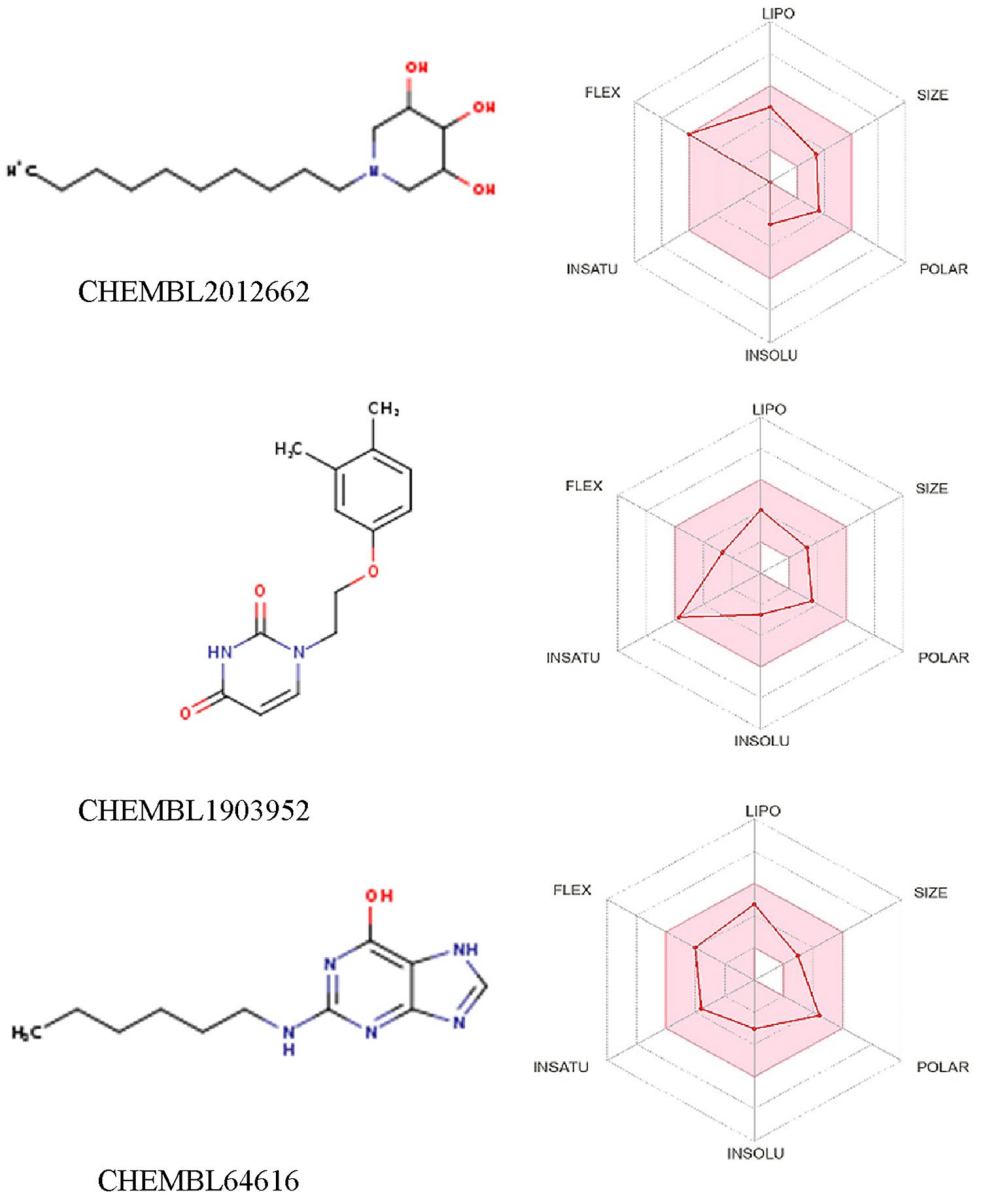
ADME profiles in web diagrams of the three final hits along with their structures. The pink area in the web diagrams are acceptable ranges for the values of lipophilicity, solubility, flexibility, unsaturation and molecular size while the white area are disallowed regions. which primarily determine the pharmacokinetic profiles of drug-like compounds.

Swiss ADME server was used to examine the pharmacokinetic and physicochemical characteristics of 127 hit compounds that were derived from the docking studies. Lipinski's rule of five, a compound's solubility, lipophilicity, flexibility, and ability to penetrate the blood–brain barrier are among the factors taken into account in this work. As a result, 27 hit compounds were chosen. All of the compounds' molecular weights were discovered to fall within the typical range of 95% of the medicines that were tested. It was discovered that every chemical complied with the Lipinski criterion of five drug likelihood with no exceptions. Based on the optimal combinations of PLIP, PLIF (Fig. S2), and ADME profiles identified, three compounds were selected. As shown in Fig. 4, the three compounds projected high oral absorption rate and moderate to well blood–brain barrier permeability. Table S1 provides a comprehensive explanation of the pharmacokinetic parameters for these compounds. In conclusion, it can be deduced that these compounds exhibit favorable ADME profiles, making them promising candidates for further exploration. Additionally, the toxicity profiles of these compounds were also calculated via Protox 3.0 webserver. The three compounds were found to be very non-toxic, representing appropriate safety profiles, as can be seen in Table S2.

### Molecular binding interactions

**Figure 5:**
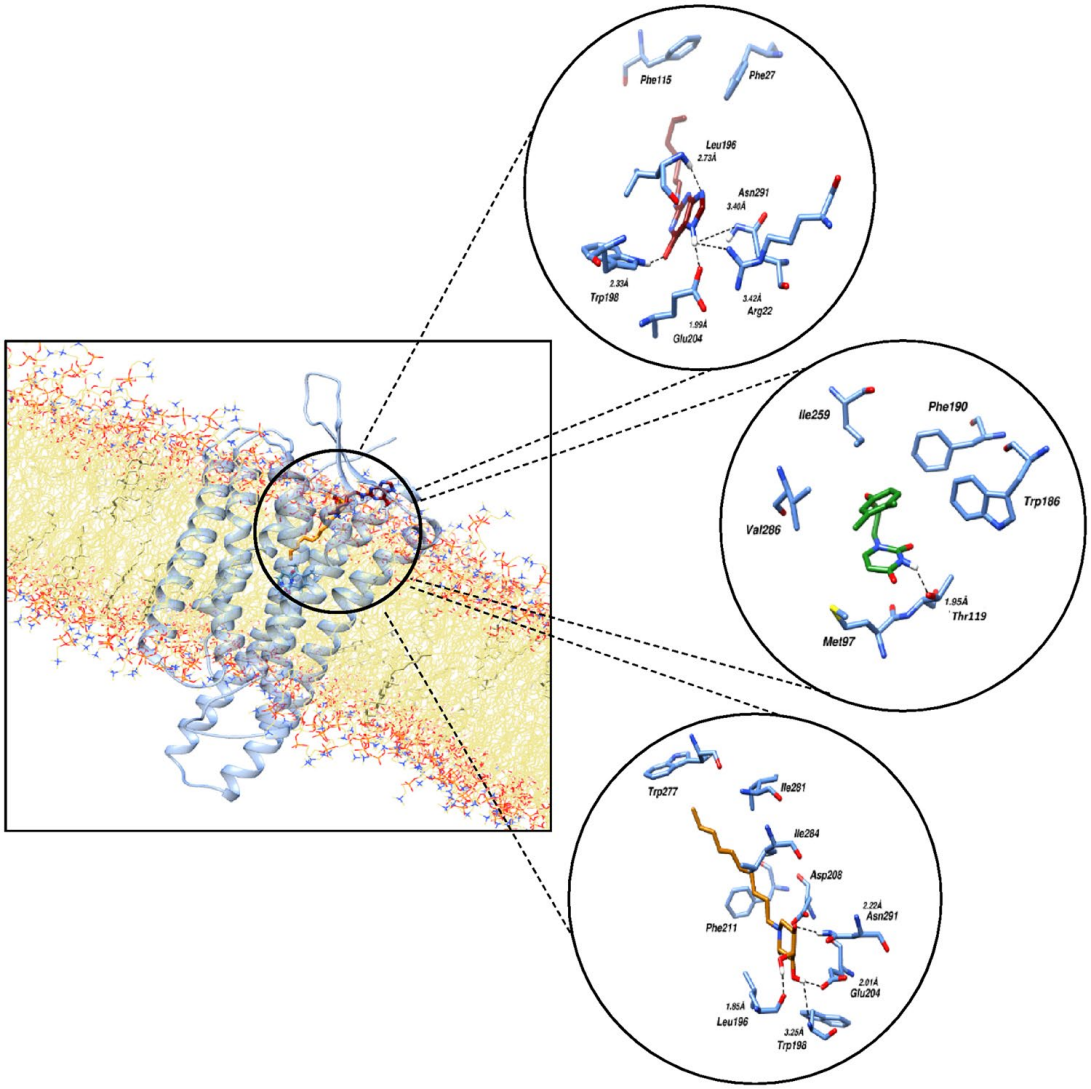
Molecular binding interactions observed after the molecular dynamic simulation of the membrane bound FFAR4 with the three compounds (Compound 1: Maroon, Compound 2: Green, Compound 3: Orange). The interacting residues are represented as sticks, while black dotted lines represent the hydrogen bonds.

In order to observe the molecular binding pattern within the complexes, the resultant trajectories of the simulations were examined with a distance limited at 4 Å. Figure 5 depicts the molecular binding interactions observed after the molecular dynamic simulation of the membrane-bound FFAR4 with compounds 1, 2, and 3. Upon examining the intermolecular interactions, it becomes apparent that the stability of protein–ligand complexes in all three simulated systems primarily relies on a multitude of hydrogen bonds.

Compounds 1 and 3 both primarily showed hydrogen bonding interactions with FFAR4 residues including Leu196, Trp198, Glu204 and Asn291 in common at a distance of 2.73, 2.33, 2.73, 2.33 Å and 1.85, 3.25, 2.01, 2.22 Å respectively. These observations are in-line with the interactions observed via molecular docking studies in our present work, as well as the previous reports^14^. Compound 1 showed an additional bonding with hydrogen atom involving Arg22 at a distance of 3.42 Å and hydrophobic interactions with residue Phe27 and Phe115, while residues Trp277, Ile261, Ile284, Phe211 showed hydrophobic interactions with compound 3. Compound 2 on the other hand only showed one hydrogen bond interaction involving Thr119 at a distance of 1.95 Å.

### Structural stability and flexibility

**Figure 6:**
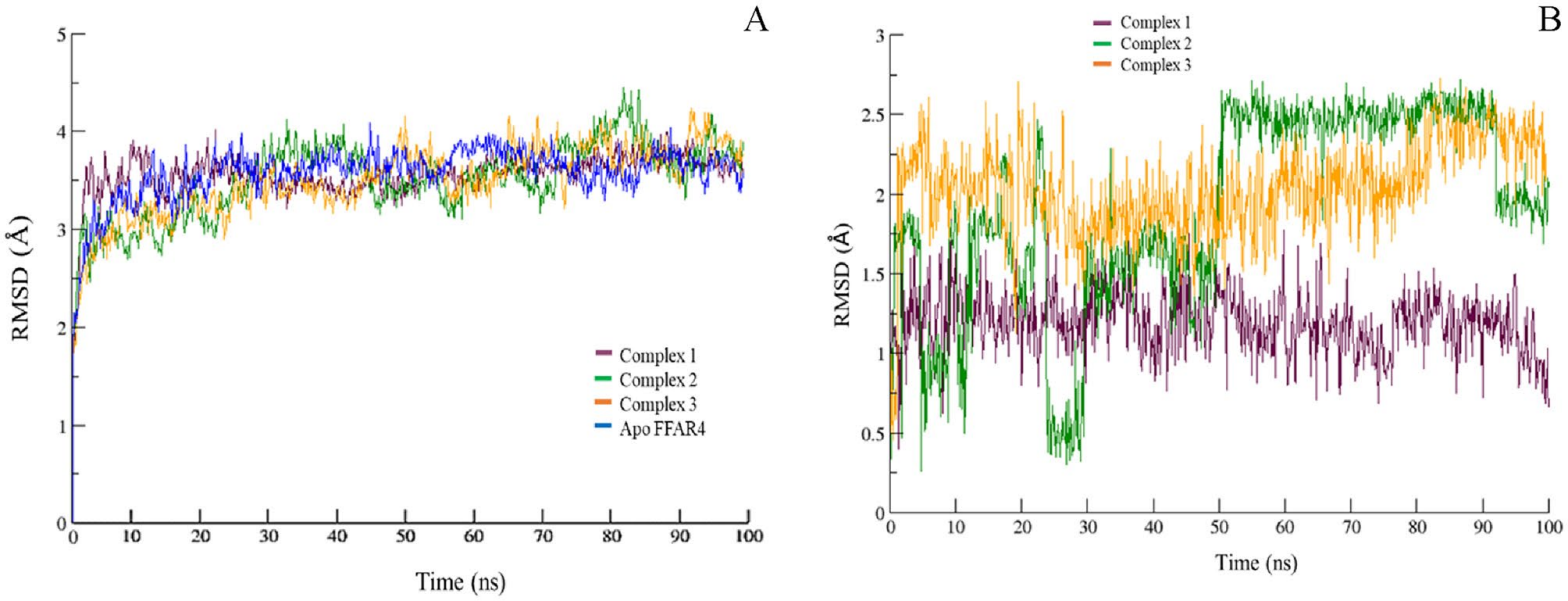
(**A**) RMSD of FFAR4 backbone carbon atoms as part of protein–ligand complex 1, 2 and, 3 compared to Apo FFAR4, for the entire duration of the simulation. (**B**) RMSD of the three compounds as part of protein–ligand complex 1, 2 and, 3 during the course of 100 ns of simulation.

To assess the structural stability, flexibility, and overall dynamics of the FFAR4 complexes, RMSDs of the 100 ns simulated trajectories were calculated and shown in Fig. 6A. The RMSD of any simulated bio molecular system serves as an essential parameter to gauge the system’s stability. In the context of structural biology when studying the atomic coordinates, the lower RMSD values indicate greater system stability whereas larger RMSD values correspond to less stable complexes^28^. Throughout the simulation, all three systems exhibited fluctuations, with consistent RMSDs. FFAR4 as part of complex 1, 2, and 3 demonstrated an average RMSD of 3.57 ± 0.23, 3.64 ± 0.46, and 3.51 ± 0.34 nm respectively, while Apo FFAR4 exhibited an average of 3.57 ± 0.29 nm deviation. As can be observed in the plot, throughout the entirety of the simulation, comparable RMSDs with minimal fluctuations were observed. Although the fluctuation varied slightly across the three complexes, they remained within the consistent range comparable with the unbound FFAR4.

Moreover, further visual analysis of the trajectories was also performed to assess the stability of the bound ligands. The ligands as part of complex 1, 2, and 3 projected an average RMSD of 1.19 ± 0.19 1.91 ± 0.61, and 2.03 ± 0.30 nm, respectively. As depicted in Fig. 6B, compound 2 exhibited the most deviations throughout the simulation, while compound 1 and 3 remained comparatively steady and showed fewer deviations, indicating stable protein–ligand complexes. During the simulations, consistent deviations in the same regions were observed for compound 1 and compound 3, indicating stable binding modes relative to their initial positions. However, the magnitude of deviations is higher in case of complex 3 as compared to others. The instability observed in case of compound 2 can be explained by the inconsistent intermolecular interactions observed (Fig. 5), suggesting instable protein–ligand complex. RMSD of FFAR4 backbone carbon atoms as part of protein–ligand complex 1, 2, 3, and reference ligand compared to Apo FFAR4 (Fig. S3), and the RMSD of the selected compounds when compared with the reference ligand (Fig. S4), indicate similar structural stability.

**Figure 7:**
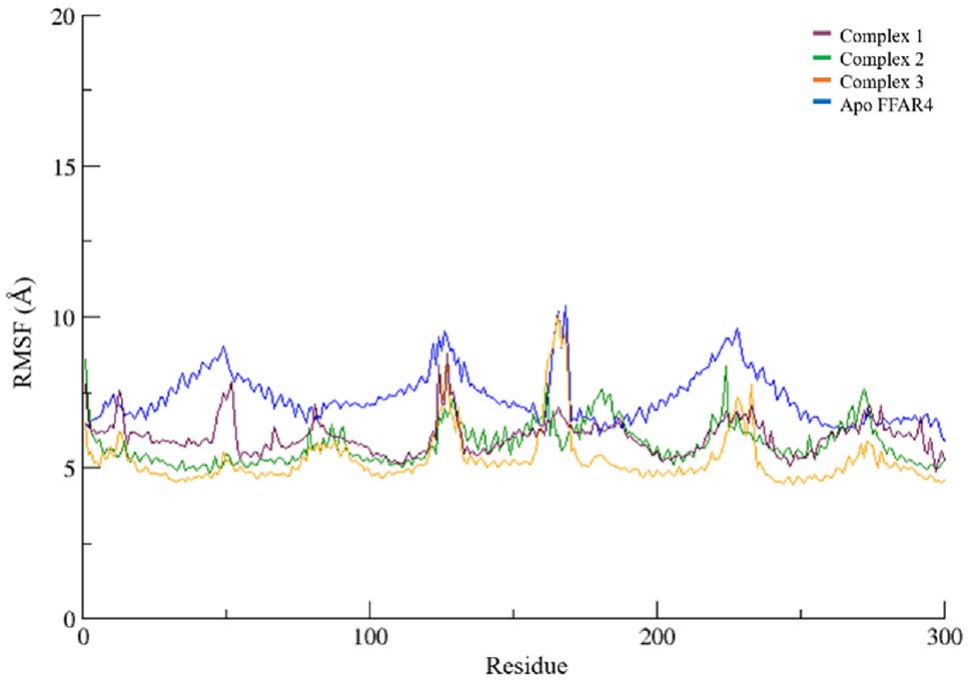
RMSF of FFAR4 backbone carbon atoms as part of protein–ligand complex 1, 2, and 3, as compared to Apo FFAR4, for the entire duration of the simulation.

In order to assess the inherent flexibility of the FFAR4 residues during the course of simulation, Root Mean Square Fluctuations (RMSF) of the simulated trajectories, were computed. The RMSF provides information regarding residue-level perturbations where higher values within a bio molecular system signify flexibility and, consequently a less stable state^29^. On the other hand, reduced fluctuation imparts greater stability to the system^29^. The average RMSF (Fig. 7) experienced by the FFAR4 residues in complex 1, 2, and 3 were noted as 6.03 ± 0.57, 5.77 ± 0.66, and, 5.27 ± 0.87 while Apo FFAR4 exhibited an average of 7.34 ± 0.82 nm. As can be observed in the plot, throughout the entirety of the simulation, comparable RMSFs with minimal fluctuations in the same regions were observed with all the complexes, with the exception of complex 1 which showed higher fluctuations. The observed fluctuations can be attributed to the residues involved in binding the ligands and forming the protein–ligand complex. The RMSF of the selected compounds when compared with the reference ligand (Fig. S3), indicates similar flexibility of the FFAR4 residues during the course of simulation. The RMSF plots of the key residues of the FFAR4 binding pocket are provided in Supplementary as Fig. S5.

### Structural compactness

**Figure 8:**
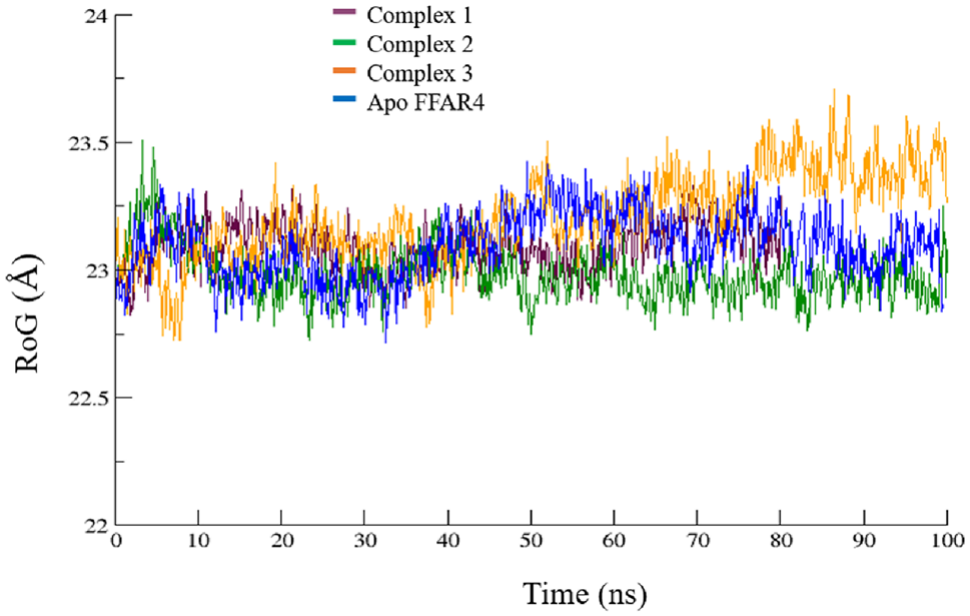
The compactness of FFAR4 as part of protein–ligand complexes 1, 2, ad 3, as compared to FFAR4, during the course of entire simulation.

The time-dependent convergence of the radius of gyration (RoG), representing the structural properties of the simulated ensembles, was further examined and illustrated in Fig. 8. RoG, reflecting the compactness of the protein, is defined as the root-mean-squared distance of the components within an object relative to its center of mass. After computing the influence of ligand binding to the receptor at molecular level, the folding behavior of the protein with respect to time was studied via RoG. A properly folded conformation in general features constant gyration values, while on the other hand, distortion in the folding behavior induces the RoG values to change over time^30^. The gyration values observed for the studied systems displayed fluctuations spanning from 22.8 to 23.5 nm highlighting the divergence in the structural compactness. The mean values of 23.04 ± 0.09, 22.98 ± 0.10, 23.20 ± 0.10, 23.09 ± 0.12, were observed for the Apo FFAR4 and the three complexes, respectively. The RoG data shows that all three complexes were structurally compact, ordered. The RoG of the selected compounds when compared with the reference ligand (Fig. S3), indicates similar compactness in case of complex 1 and 3, while complex 2 showed fluctuations.

### Conformational motions and thermodynamic stability

To capture the substantial conformational changes with respect to free energy, PCA and FEL were computed for all of the simulated systems. We generated 2D FEL plots for both the ligand-free state and ligand-bound complex, utilizing principal component values with a color-coded contour map indicating energy levels and simulation extremes. All three systems demonstrated different conformational patterns with various energy basins.

**Figure 9:**
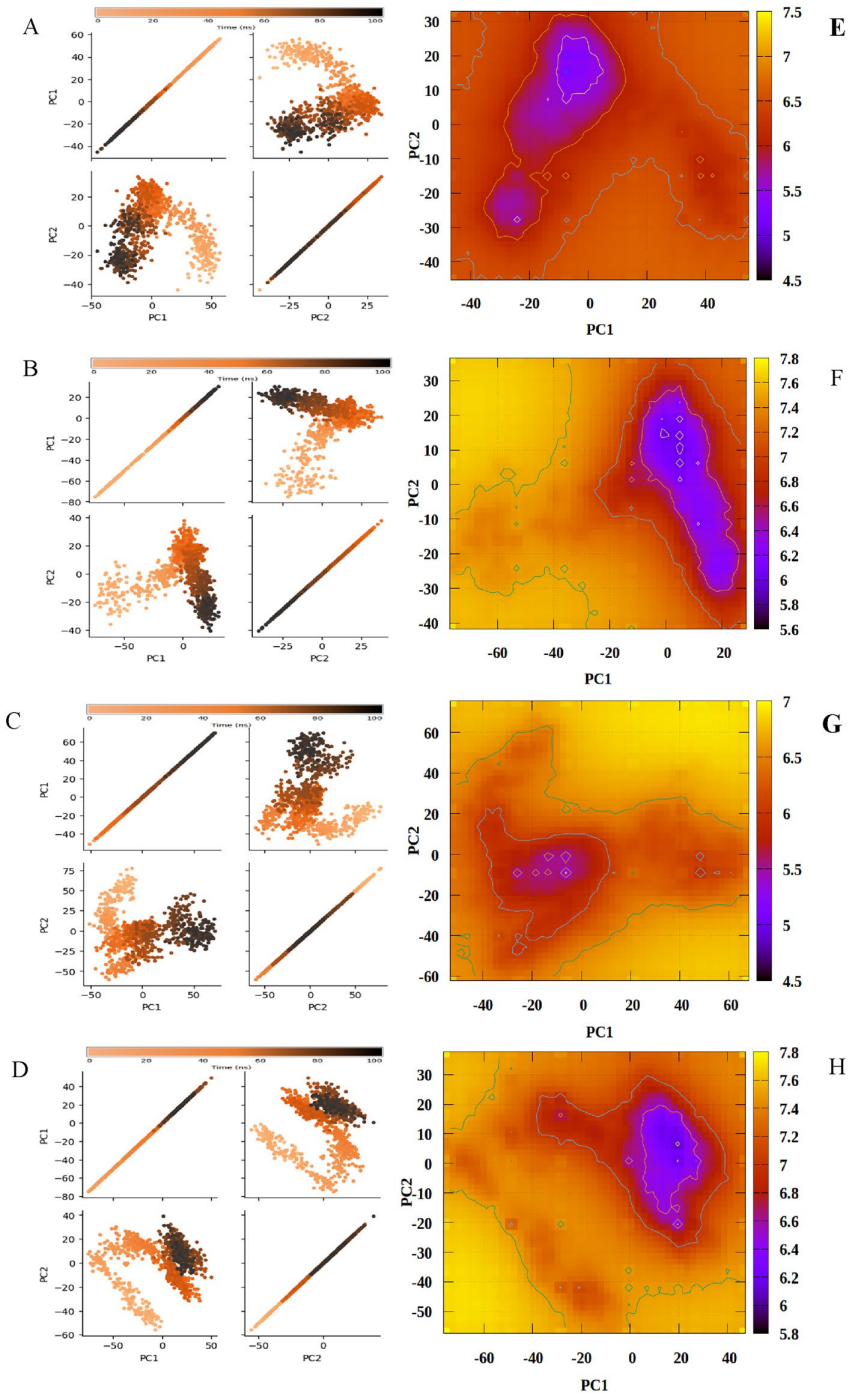
The Conformational PCA plots computed from the backbone atoms of (**A**) Apo FFAR4. (**B**) Complex 1 (**C**) Complex 2 (**D**) Complex 3 next to the corresponding Free energy landscapes (FELs) of (**E**) Apo FFAR4 (**F**) Complex 1 (**G**) Complex 2 (**H**) Complex 3.

In general, all of the systems demonstrated different conformational patterns with various energy basins. In Apo state, the membrane bound receptor protein showed a high degree of variance in PC1 and PC2 during both the initial and final MD conformations suggesting instability. As can be observed in Fig. 9A, initially, the PCs values decrease but towards the end of the 100 ns simulation an increase can be observed. Conversely, the stability of the protein backbone in complex 1 is evident from much smaller values of PC1 and PC2 towards the end of simulations as can be seen in Fig. 9B as compared to Apo in which initial variances of PC1 and PC2 begin at -40 and -50 respectively. Complex 2 also demonstrates much smaller values at − 10 and − 40 as seen in Fig. 9C. For complex 3, the final steps show PC1 and PC2 variances at 20 and 40 again indicating a more stabilized complex as seen in Fig. 9D. It can be inferred from the PCA plots, that all three compounds confer stability to the complex which is in-line with the RMSD observations.

The FEL plot of the Apo FFAR4 show the best free energy profiles at around PC1 and PC2 variances. As can be observed in Fig. 9E, the Apo FFAR4 shows fewer high free energy basins, as compared to the protein–ligand complexes as seen in Figs. 9F–H which show higher free energies and lower variances, which can be attributed to the binding of the ligand. The highest free energy states for the first compound complex lies in in range of 10 to 0 (PC1) and 20 to 0 (PC2) Fig. 9F. In case of the compound 2 these values are − 2 to − 22 (PC1) and − 5 to 0 (PC2) Fig. 9G, while for the third complex, FEL contour hovers around 20 to -5 (PC1) and 20 to − 10 (PC2) Fig. 9H. As can be observed via the FEL plots, the contours shift to wider energy basins, inferring stable protein–ligand complex.

## Conclusion

In this study, we utilized structure-based machine learning techniques that rely on molecular fingerprinting to screen compounds as potential FFAR4 agonists. This approach involves encoding molecular structures into bit arrays using specific criteria, known as molecular fingerprinting. These bit arrays represent the structural features of the molecules, which are then processed by machine learning algorithms to identify patterns among the selected compounds. To train our machine learning model, we utilized a training set composed of derived fingerprints from the structures of known FFAR4 activators, inhibitors, and a decoy dataset. The fingerprints were encoded into bit arrays, and the Bayesian network algorithm was employed to calculate the posterior probability of values appearing in the training dataset. This allowed us to draw conclusions about the structural requirements for a compound to act as an activator. Subsequently, the trained model was applied to filter compounds from a large screening dataset. The compounds that passed this initial screening were subjected to ADME and Toxicity prediction, Molecular Docking, and post-docking analysis to verify their binding affinities and binding poses. Additionally, Molecular Dynamics simulations of the membrane-bound FFAR4-ligand complexes were conducted to assess the stability of the human FFAR4 with the selected compounds.

Comprehensive molecular analyses, including investigations into protein–ligand binding interactions, RMSD, RMSF, RoG, PCA, and FEL, were performed. These analyses provided insights into the interaction between the identified compounds (1 and 3) and critical residues of FFAR4, corroborating previous reports. When compared to the Apo FFAR4, RMSD and RMSF analyses indicated minimal structural deviation and fluctuations for these compounds. Furthermore, RoG measurements suggested the formation of stable complexes between these compounds and FFAR4. PCA and FEL analyses yielded similar results. Collectively, these findings enhance our understanding of the functions and mechanisms of FFAR4, while also highlighting compounds CHEMBL2012662 and CHEMBL64616 as potential FFAR4 agonists. These compounds show promise as innovative treatments for metabolic and immune-related conditions. Further investigation into their potential is warranted to explore their therapeutic value. By deepening our comprehension of FFAR4's functions and mechanisms, these findings shed light on the development of novel treatments for metabolic and immune-related diseases.

### Supplementary Information

Supplementary Information.
