# Supplementary material for: Predicting FFAR4 agonists using structure-based machine learning approach based on molecular fingerprints
Source: Sci Rep. 2024 Apr 24;14:9398. doi: 10.1038/s41598-024-60056-z (PMC11043068; doi:10.1038/s41598-024-60056-z)
Supplement: Supplementary file 1 — Supplementary Information. [file 41598_2024_60056_MOESM1_ESM.docx]

**Predicting FFAR4 Agonists Using Structure-Based Machine Learning Approach Based On Molecular Fingerprints.**

**Supplementary Information**

Zaid Anis Sherwani^a^, Syeda Sumayya Tariq^a^, Mamona Mushtaq^a^, Ali Raza Siddiqui^b^, Mohammad Nur-e-Alam^c^, Aftab Ahmad^d^, Zaheer Ul-Haq^a*^

# ^a^ Dr. Panjwani Center for Molecular Medicine and Drug Research, International Center for Chemical and Biological Sciences, University of Karachi, Karachi-75270, Pakistan;

# ^b^ H.E.J Research Institute of Chemistry, International Center for Chemical and Biological Sciences, University of Karachi, Karachi-75270, Pakistan

^c^ Department of Pharmacognosy, College of Pharmacy, King Saud University, P.O. Box. 2457, Riyadh 11451, Kingdom of Saudi Arabia

^d^ Department of Biomedical and Pharmaceutical Sciences, Chapman University School of Pharmacy, Irvine, CA, 92618, USA

**Fig. S1:** Methodology Flowchart

**Structure Based Machine Learning**

Training Set

Created by obtaining compounds from ChEMBL database as Actives, Inactives, Randoms, and Decoys

Machine Learning Model

Based on: Bayesian Network Algorithm

Model Validation: Performance Metrics

Morgan Fingerprints of the training set compounds calculated via CHEMDES server

Test Set

 Derived by applying Lipinski’s Rule of 5 and computing the Morgan Fingerprints for all compounds within the ChEMBL database, narrowing down 6 million compounds to 32,000

Application of Machine Learning Model identified 693 compounds as Actives from total of 32000.

**Molecular Docking**

The 693 active compounds identified were subjected to docking against the FFAR4 structure retrieved from RCSB PDB, following established protocols.

Post – Docking Analyses

The binding poses of the top-ranked compounds were examined using: PLIF, PLIP, identifying 127 compounds for further investigation.

**Utilizing Machine Learning Model to screen for potential FFAR4 agonists**

**Molecular Dynamic Simulation**

Membrane Simulations of FFAR4 with the selected compounds was carried out for 100ns each, using established Molecular Dynamic Simulation protocols.

Post – MD Simulation Analyses: Evaluation of the obtained trajectories based on RMSD, RMSF, Rg, PCA, FEL

Two compounds put forth as as potential FFAR4 agonists

ADME Profiling led to the identification of 27 compounds to be considered for further studies.

Based on the best recorded PLIP, PLIF, and ADME profiles, three compounds were ultimately selected for in-depth MD Simulations studies.

**
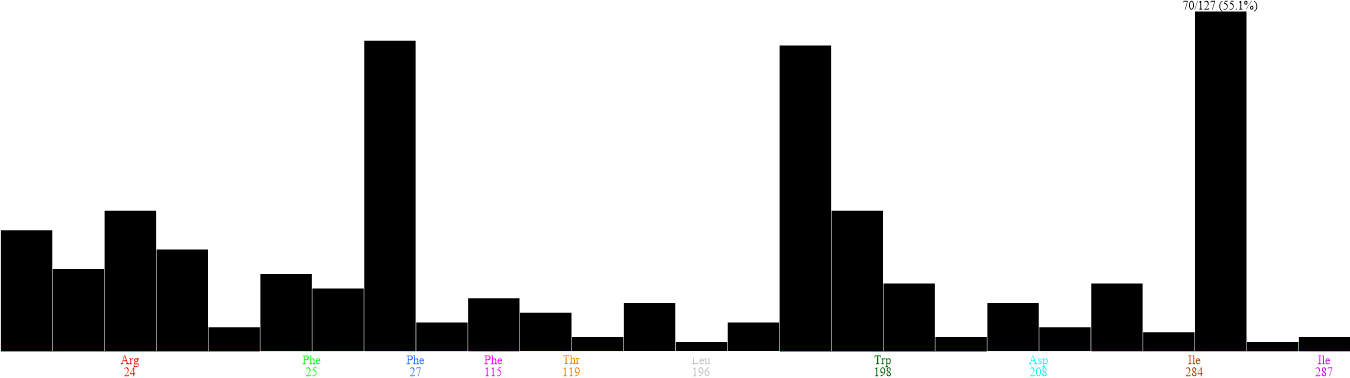
**

**
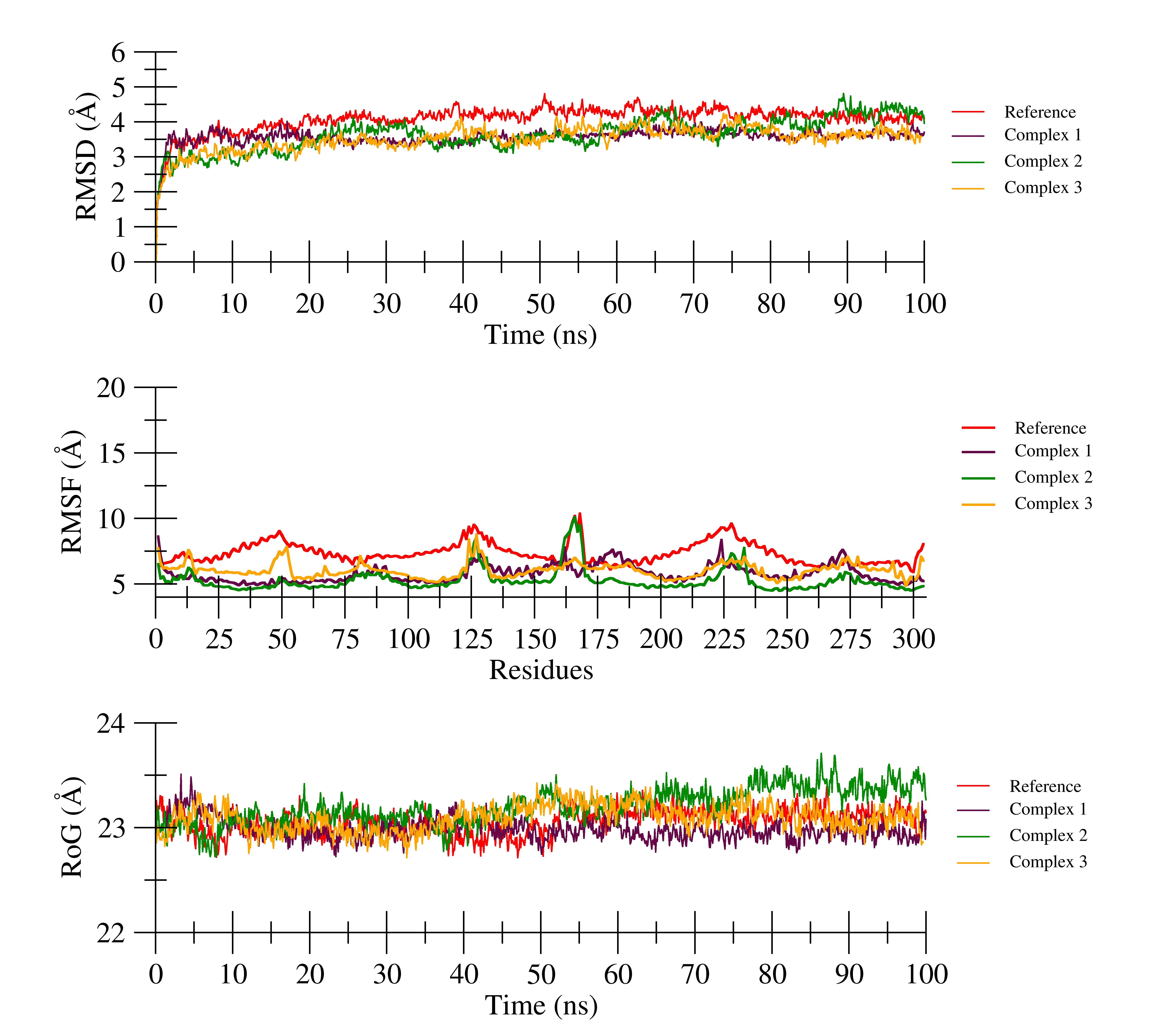
Fig. S2:** PLIF (Protein–Ligand Interaction Fingerprints) of the 127 shortlisted hits.

**Fig. S3:** RMSD, RMSF, Rg of FFAR4 with the control ligand.

**
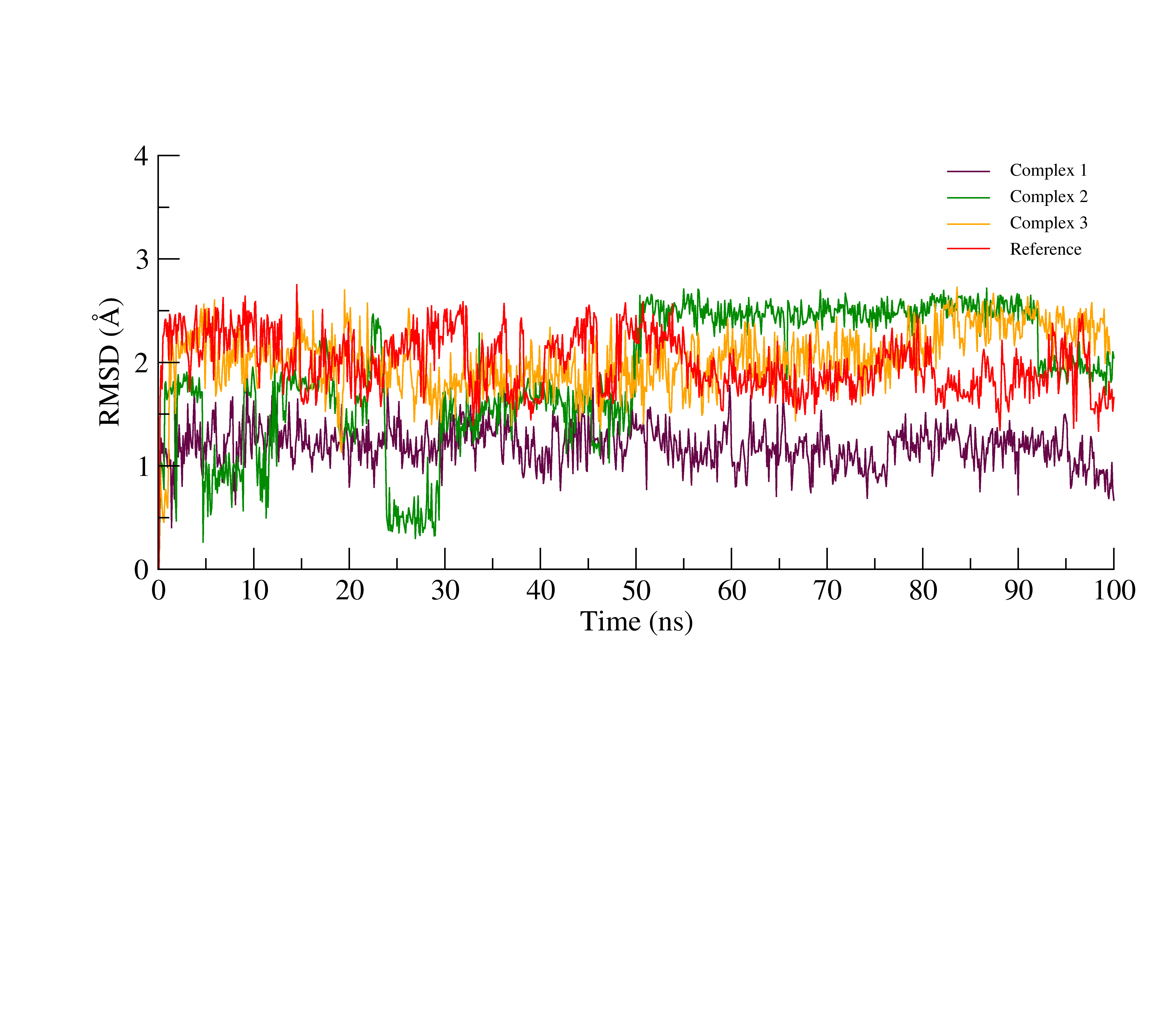
**

**Fig. S4:** The RMSF plots of the selected compounds when compared with the reference ligand.


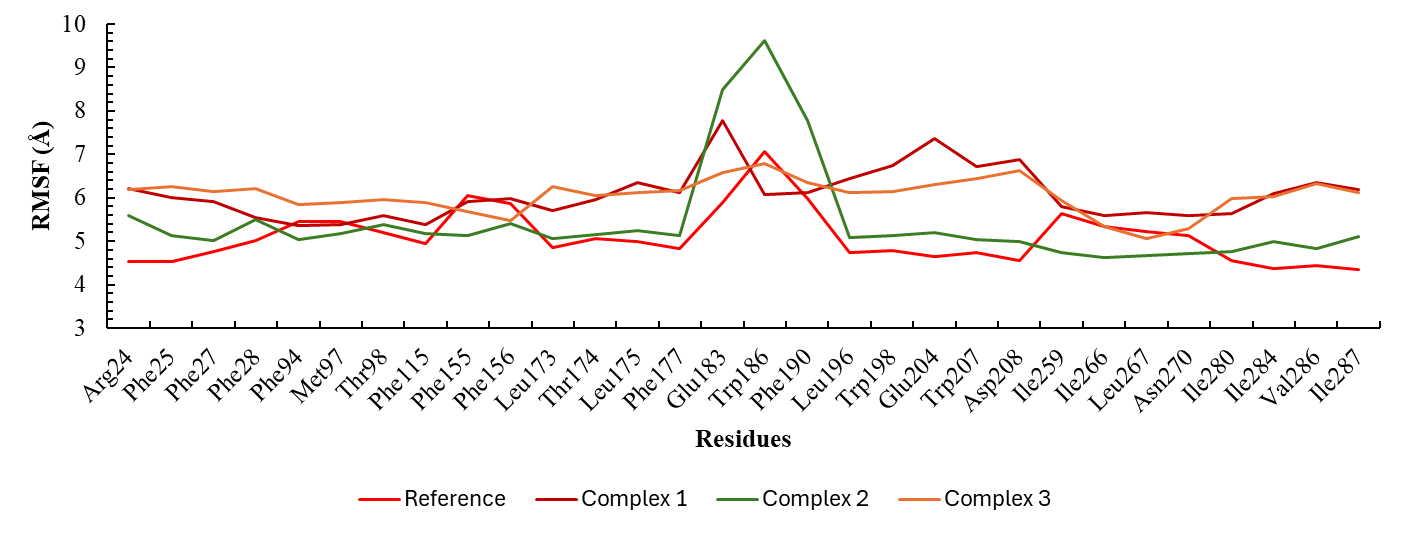


**Fig. S5:** The RMSF plots of the key residues of the FFAR4 binding pocket.

**Table S1:** Detailed ADME properties of the three shortlisted hits predicted via SWISS ADME.

| CHEMBL ID | 2012662 | 1903952 | 64616 | |
| --- | --- | --- | --- | --- |
| Physicochemical properties | | | | |
| MW | 273.41 | 260.29 |  | |
| No. of Heavy atoms | 19 | 19 | 17 | |
| No. of Aromatic heavy atoms | 0 | 12 | 9 | |
| Fraction Csp3 | 1.00 | 0.29 | 0.55 | |
| No. of Rotatable bonds | 9 | 4 | 6 | |
| No. of H-bond acceptors | 4 | 3 | 4 | |
| No. of H-bond donors | 3 | 1 | 3 | |
| MR | 82.40 | 73.34 |  | |
| TPSA | 63.93 | 64.09 | 86.72 | |
| Lipophilicity | | | |  |
| iLOGP | 2.96 | 2.44 | 1.97 | |
| XLOGP3 | 2.68 | 1.61 | 2.73 | |
| WLOGP | 1.14 | 1.23 | 1.86 | |
| MLOGP | 1.15 | 1.60 | 1.02 | |
| Silicos-IT Log P | 1.96 | 2.87 | 1.84 | |
| Consensus Log P | 1.98 | 1,95 | 1.88 | |
| Water Solubility | | | |  |
| ESOL Log S | -2.63 | -2,67 | -3.02 | |
| ESOL Solubility (mg/ml) | 6.42E-01 | 5.55E-01 | 2.22E-01 | |
| ESOL Solubility (mol/l) | 2.35E-03 | 2.13E-03 | 9.67E-04 | |
| ESOL Class | Soluble | Moderately soluble | Soluble | |
| Ali Log S | -3,67 | -4.49 | -4.21 | |
| Ali Solubility (mg/ml) | 5.78E-02 | 7.04E-01 | 1,47E-02 | |
| Ali Solubility (mol/l) | 2.11E-04 | 2,71E-03 | 6,23E-05 | |
| Ali Class | Soluble | Soluble | Moderately  Soluble | |
| Silicos-IT LogSw | -2.22 | -4.49 | -4.06 | |
| Silicos-IT Solubility (mg/ml) | 1,66E+00 | 8.50E-03 | 2.04E-02 | |
| Silicos-IT Solubility (mol/l) | 6.05E-03 | 3.27E-05 | 8.69E-05 | |
| Silicos-IT class | Soluble | Moderately  Soluble | Moderately soluble | |
| Pharmacokinetics | | | | |
| GI absorption | High | High | High | |
| BBB permeant | Yes | No | No | |
| Pgp substrate | No | No | Yes | |
| CYP1A2 inhibitor | No | Yes | Yes | |
| CYP2C19 inhibitor | No | No | No | |
| CYP2C9 inhibitor | No | No | No | |
| CYP2D6 inhibitor | No | No | No | |
| CYP3A4 inhibitor | No | No | No | |
| log Kp (cm/s) | -6.07 | -6.74 | -5.80 | |
| Druglikeness | | | |  |
| s | 0 | 0 | 0 | |
| No. of Ghose violations | 0 | 0 | 0 | |
| No. of Veber violations | 0 | 0 | 0 | |
| No. of Egan violations | 0 | 0 | 0 | |
| No. of Muegge violations | 0 | 0 | 0 | |
| Bioavailability Score | 0.55 | 0.55 | 0.55 | |
| Medicinal Chemistry | | | |  |
| No. of PAINS alerts | 0 | 0 | 0 | |
| No. of Brenk alerts | 0 | 0 | 0 | |
| Leadlikeness | 1 Viol | Yes | 1 Viol | |
| Synthetic Accessibility | 3,53 | 2,26 | 2.44 | |

**Table S2:** Toxicity profiles of the shortlisted compounds estimated by *in silico* approach.

I: inactive, A: active, the values given in brackets adjacent to the I & A marks represent the probability scores.

| Compound | Toxicity class | LD_50_ (mg/kg) | Hepatotoxicity | Carcinogenicity | Immunotoxicity | Mutagenicity | Cytotoxicity |
| --- | --- | --- | --- | --- | --- | --- | --- |
| CHEMBL-2012662 | 4 | 1370 | I (0.94) | I (0.57) | I (0.87) | I (0.78) | I (0.80) |
| CHEMBL-1903952 | 5 | 2140 | I (076) | I (0.66) | I (0.95) | I (0.78) | I (0.78) |
| CHEMBL-64616 | 4 | 1759 | I (0.76) | I (0.58) | A (0.87) | I (0.59) | I (0.76) |

**Table S3:** H bond occupancy

| Reference Ligand + Receptor | | | |
| --- | --- | --- | --- |
| #Acceptor | Donor H | Donor | Occupancy |
| Reference_Ligand@O2 | PHE_25@HN | PHE_25@N | 40.1 |
| Reference_Ligand@O3 | ARG_22@HH11 | ARG_22@NH1 | 39.7 |
| Reference_Ligand@O2 | ARG_22@HH11 | ARG_22@NH1 | 27.1 |
| Reference_Ligand@O3 | PHE_25@HN | PHE_25@N | 26.9 |
| Reference_Ligand@O3 | ARG_24@HH22 | ARG_24@NH2 | 13.3 |
| THR_119@OG1 | Reference_Ligand@H35 | Reference_Ligand@O1 | 4.3 |
| Reference_Ligand@O2 | ARG_22@HE | ARG_22@NE | 2 |
| Reference_Ligand@O2 | ASN_291@HD22 | ASN_291@ND2 | 1.9 |
| Reference_Ligand@H33 | ASN_291@HD22 | ASN_291@ND2 | 1.2 |
| Reference_Ligand@H32 | ASN_291@HD22 | ASN_291@ND2 | 0.9 |
| Reference_Ligand@O2 | THR_195@HG1 | THR_195@OG1 | 0.9 |
| Reference_Ligand@O2 | LEU_196@HN | LEU_196@N | 0.8 |
| Reference_Ligand@O3 | ASN_291@HD22 | ASN_291@ND2 | 0.6 |
| Reference_Ligand@O3 | ARG_22@HE | ARG_22@NE | 0.5 |
| Reference_Ligand@H12 | ASN_291@HD22 | ASN_291@ND2 | 0.5 |
| Reference_Ligand@O3 | THR_195@HG1 | THR_195@OG1 | 0.5 |
| Reference_Ligand@O1 | THR_119@HG1 | THR_119@OG1 | 0.5 |
| Complex 1 | | | |
| GLU_204@OE1 | Compound_1@H17 | Compound_1@N5 | 38.7 |
| GLU_204@OE2 | Compound_1@H17 | Compound_1@N5 | 29.6 |
| Compound_1@O | TRP_198@HE1 | TRP_198@NE1 | 15.7 |
| Compound_1@N4 | THR_195@HG1 | THR_195@OG1 | 2.7 |
| Compound_1@N4 | LEU_196@HN | LEU_196@N | 2.5 |
| LEU_196@O | Compound_1@H1 | Compound_1@O | 1.4 |
| Compound_1@N3 | ASN_291@HD22 | ASN_291@ND2 | 1 |
| Compound_1@N4 | ASN_291@HD22 | ASN_291@ND2 | 0.9 |
| LEU_288@O | Compound_1@H1 | Compound_1@O | 0.6 |
| Complex 2 | | | |
| THR_98@OG1 | Compound_2@H5 | Compound_2@N2 | 13.2 |
| GLU_183@OE2 | Compound_2@H5 | Compound_2@N2 | 6.5 |
| Compound_2@O2 | TRP_177@HE1 | TRP_177@NE1 | 5.9 |
| GLU_183@OE1 | Compound_2@H5 | Compound_2@N2 | 5.8 |
| Compound_2@O3 | ARG_3@HH12 | ARG_3@NH1 | 2.1 |
| Compound_2@O2 | THR_98@HG1 | THR_98@OG1 | 1.8 |
| Compound_2@O3 | ASN_270@HD22 | ASN_270@ND2 | 0.9 |
| MET_97@O | Compound_2@H5 | Compound_2@N2 | 0.5 |
| Complex 3 | | | |
| ASP_208@OD2 | Compound_3@HN | Compound_3@N | 66.16 |
| GLU_204@OE2 | Compound_3@H1 | Compound_3@O1 | 13.76 |
| ASP_208@OD1 | Compound_3@HN | Compound_3@N | 12.35 |
| GLU_204@OE1 | Compound_3@H4 | Compound_3@O2 | 10.54 |
| LEU_196@O | Compound_3@H1 | Compound_3@O1 | 9.54 |
| GLU_204@OE2 | Compound_3@H4 | Compound_3@O2 | 6.53 |
| GLU_204@OE2 | Compound_3@H31 | Compound_3@O3 | 5.02 |
| Compound_3@H4 | TRP_198@HN | TRP_198@N | 2.71 |
| GLU_204@OE1 | Compound_3@H1 | Compound_3@O1 | 2.21 |
| Compound_3@H31 | ASN_291@HD22 | ASN_291@ND2 | 2.21 |
| GLU_204@O | Compound_3@H4 | Compound_3@O2 | 1.51 |
| ASP_208@OD1 | Compound_3@H31 | Compound_3@O3 | 1.31 |
| GLU_204@O | Compound_3@H31 | Compound_3@O3 | 1.1 |
| Compound_3@O3 | ASN_291@HD21 | ASN_291@ND2 | 1 |
| TRP_207@O | Compound_3@H4 | Compound_3@O2 | 0.7 |
| Compound_3@H31 | ASN_291@HD21 | ASN_291@ND2 | 0.6 |

-------------------------------------
